# Degradation Behaviors of Polylactic Acid, Polyglycolic Acid, and Their Copolymer Films in Simulated Marine Environments

**DOI:** 10.3390/polym16131765

**Published:** 2024-06-21

**Authors:** Zeyu Chen, Xi Zhang, Ye Fu, Yujuan Jin, Yunxuan Weng, Xinchao Bian, Xuesi Chen

**Affiliations:** 1 College of Light Industry Science and Engineering, Beijing Technology and Business University, Beijing 100048, China; c1554192404@163.com (Z.C.); fuye@btbu.edu.cn (Y.F.); jinyujuan@th.btbu.edu.cn (Y.J.); 2 Changchun Institute of Applied Chemistry, Chinese Academy of Sciences, Changchun 130022, China; xcbian@ciac.ac.cn (X.B.); xschen@ciac.ac.cn (X.C.)

**Keywords:** PLA, PGA, PLGA, seawater environment, degradation

## Abstract

Poly(lactic acid) (PLA) and poly(glycolic acid) (PGA) are extensively studied biodegradable polymers. However, the degradation behavior of their copolymer, poly(lactic-co-glycolic acid) (PLGA), in marine environments has not yet been confirmed. In this study, the changes in macroscopic and microscopic morphology, thermal properties, aggregation, and chemical structure of PLA, PGA, PLGA-85, and PLGA-75 (with 85% and 75% LA content) in simulated marine environments were investigated. Results revealed that degradation occurred through hydrolysis of ester bonds, and the degradation rate of PGA was faster than that of PLA. The amorphous region degraded preferentially over the crystalline region, leading to cleavage-induced crystallization and decreased thermal stability of PLA, PLGA-85, and PLGA-75. The crystal structures of PLGAs were similar to those of PLA, and the higher GA content, the faster was the degradation rate. This study provides a deeper understanding of the seawater degradation behaviors of PLA, PGA, and their copolymers, and provides guidance for the preparation of materials with controllable degradation performance.

## 1. Introduction

Over the past thirty years, there has been a significant increase in the production and usage of synthetic polymers [1,2,3]. These polymers have a profound impact on engineering and commercial applications due to their stability towards chemicals, hydrolysis, temperature, light, microorganisms, and other factors [4]. However, this inherent characteristic of polymers poses a challenge to society in managing plastic waste that enters the environment [5,6]. According to statistics, currently, humans produce approximately 430 million tons of plastic annually, two-thirds of which quickly become waste. Each year, 19 to 23 million tons of plastic waste flow into aquatic ecosystems, particularly marine environments, causing significant harm to aquatic ecosystems [7,8]. Therefore, it is imperative to find reliable methods to address marine plastic pollution. To achieve sustainable long-term development, starting from the material itself and researching plastic materials that can rapidly self-degrade in seawater is the most fundamental and effective approach to solving this problem [9,10,11].

Biodegradable polymer materials are an important type of synthetic biomaterials that are widely used in medical fields such as trauma repair and tissue regeneration [12,13,14]. Among the developed biodegradable polymers, aliphatic polyesters have attracted attention due to their good melt processability, mechanical strength, and excellent degradability. Poly(lactic acid) (PLA), poly(glycolic acid) (PGA), and their copolymer poly(lactic-co-glycolic acid) (PLGA) are typical representatives of aliphatic polyesters, which are widely applied in bone tissue engineering scaffolds, drug release systems, surgical sutures, and other fields due to their good biocompatibility and controllable degradation [15,16,17,18,19].

PLA is renewable aliphatic polyester resultant from the lactic acid monomer unit (LA). It is a semi-crystalline polymer with a glass transition temperature (T_g_) of 55 °C and a melting point (T_m_) of 165 °C [20]. It possesses several excellent properties, such as strength, stiffness, biocompatibility, thermoplasticity, monomer recyclability, and good processability [21,22,23,24]. However, PLA exhibits weak nucleation ability and a low crystallization rate under homogeneous conditions. Conventional processing methods produce products with low crystallinity or even in an amorphous state, leading to a significant reduction in mechanical and thermal resistance properties. Additionally, PLA has poor hydrophilicity and long degradation cycles, which partially limit its applications [25,26,27]. PGA is another semi-crystalline, biodegradable polyester with excellent mechanical strength and barrier properties. The gas barrier property of PGA is 1000 times higher than PLA and 100 times higher than PET. It can completely degrade within 1–3 months. However, it also has disadvantages such as difficulties in processing and insufficient toughness [28,29,30,31].

PLGA, synthesized through direct condensation of lactic acid and glycolic acid or ring-opening polymerization of lactide and glycolide, can fully utilize the advantages of PLA and PGA, such as good biodegradability, biocompatibility, outstanding mechanical properties, thermal resistance, and controllable degradation [32]. Shuai et al. [33] incorporated PGA into hydroxyapatite/poly l-lactic acid (HAP/PLLA) scaffolds, which accelerated the degradation rate of the HAP/PLLA scaffolds, improved the hydrophilicity of the scaffolds, and fully demonstrated the bioactivity and osteoconductivity of HAP. Miller et al. [34] studied the difference in degradation rates between pure PLA, PGA, and their copolymers with different monomer ratios. A comparison was made between PLGAs with LA/GA ratios of 75:25, 50:50, and 25:75, as well as pure PGA. Samples were extracted from implanted bones and soft tissues of rats at 1, 2, 3, 5, 7, 9, and 11 months for testing. The results indicated that the addition of PLA slowed down the degradation rate of PGA, and the higher the proportion of PLA, the lower was the degradation rate of the copolymer. However, most studies on the degradation behavior of PLGA have been conducted in the human body fluid environment, with limited research on its degradation behavior in the marine environment.

This study investigated the degradation behavior of PLA, PGA, and PLGA with different monomer ratios in marine environments. Considering that most plastic waste in marine environments is located in coastal areas and biodegradable polymers have a higher density than seawater, they sink into the seabed after being immersed and penetrated with seawater. Therefore, the biodegradation of materials in coastal marine areas can be simulated as aerobic biodegradation at the seawater/sediment interface (intertidal zone or coastal zone) [35]. Based on this, a simulated marine environment degradation model was constructed to examine the variations in macro and micro-morphology, thermal properties, aggregation state, and chemical structure, as well as degradation rate.

## 2. Experimental

### 2.1. Materials

The thin film samples of PLA, PGA, and their copolymers used in this experiment were prepared using Shenhua Corporation’s (Beijing, China) raw materials through a solvent casting process with a thickness of 0.25 ± 0.05 mm and were placed in a 40 °C vacuum oven for 24 h to remove organic solvents. PLA (*M_n_* = 76,344) and PGA represent the pure samples of PLA and PGA. PLGA-x (x = 85 or 75) (PLGA-85: *M_n_* = 98,994; PLGA-75: *M_n_* = 42,022) represents the PLGA copolymers, where x is the mass fraction of LA in the copolymer.

The collected sediment below the low-water line from Ningbo–Zhoushan Port, Zhejiang was stored at 4 °C and used within 4 weeks after sampling. The total organic carbon (TOC), pH value, and nitrogen content of the sediment were 0.77 mg/g, 8.0, and 0.16 mg/L, respectively. Artificial seawater with a salinity of 34.0 PSU was used, and marine organisms, sea anemones, and clownfish were introduced into the simulated environment. Meanwhile, algae were not removed.

### 2.2. Biodegradation in Simulated Marine Environment

The biodegradation experiments with different thin films were conducted in a simulated marine environment at 25 °C. The film samples were cut into 5 cm × 5 cm sizes and placed at the interface between seawater and sediment. Samples were collected periodically for testing.

### 2.3. Characterization

#### 2.3.1. SEM

The surface microstructure of the samples during degradation was observed using a scanning electron microscope (SEM, Quanta FEG, FEI, Eindhoven, The Netherlands) under an acceleration voltage of 10 kV. Before the measurement, a gold layer was uniformly sputtered on the surface of the samples.

#### 2.3.2. TGA

Thermogravimetric analysis (TGA) (TA Universal V4.5A, TA Instruments, New Castle, DE, USA) was used to characterize the thermal decomposition mass loss of the samples. Samples of approximately 5–10 mg were assayed in an aluminum crucible and tested at temperatures ranging from 40 °C to 500 °C under a nitrogen atmosphere, with a heating rate of 20 °C/min.

#### 2.3.3. DSC

The thermal properties of the degraded sample were obtained using a Q100 instrument (TA Instruments, New Castle, DE, USA) through differential scanning calorimetry (DSC). Samples of about 5–8 mg were sealed in an aluminum crucible and were then heated from room temperature to 250 °C at a rate of 10 °C/min and held at a constant temperature for 5 min to eliminate thermal history. Then, they were cooled to 0 °C at a rate of 10 °C/min under a nitrogen atmosphere and re-heated to 250 °C at a heating rate of 10 °C/min.

#### 2.3.4. XRD

The crystalline structure before and after degradation was measured via X-ray diffraction (XRD, Rigaku Smart Lab SE, Tokyo, Japan) at a scanning speed of 5°/min and a scanning angle range of 10~50°. The crystallinity was estimated using the following formula:

(1) $$X_{c}=\frac{A_{c}}{A_{c}+A_{a}}$$

where *A_c_* and *A_a_* are the areas of crystalline and amorphous regions, respectively. The interplanar distance (d*_hkl_*) was calculated using Bragg’s law:

(2) $$d_{hkl}=\frac{\lambda }{2\sin\theta }$$

where *λ* represents the wavelength of X-rays and *θ* represents the diffraction angle.

#### 2.3.5. FTIR

Fourier-transform infrared spectroscopy (FTIR) analysis was conducted using an ATR-FTIR spectrometer (Thermos Fisher Scientific, Waltham, MA, USA) with a scanning range from 4000 cm^−1^ to 400 cm^−1^ at room temperature. The resolution was set at 8 cm^−1^ and each spectrum was scanned 32 times.

#### 2.3.6. XPS

Chemical elements on the surface of the thin films were investigated using X-ray photoelectron spectroscopy (XPS) on K-ALPHA^+^ with an Al K_α_ X-ray source (Thermos Fisher Scientific, Waltham, MA, USA).

#### 2.3.7. GPC

Changes in molecular weight and polydispersity index during the degradation process were tested using gel permeation chromatography (GPC-20A, Shimadzu Corporation, Kyoto, Japan) with tetrahydrofuran as the mobile phase, and calibration was performed using polystyrene standards. The sample concentration was 0.2 mg/mL, and the corresponding injection volume was 10 μL. After the samples were completely dissolved, the test was performed with the chromatographic column and detector running at 25 °C and the flow rate set to 2 mL/min. The chain scission concentration in the amorphous phase was calculated using the following equation [36]:

(3) $$S_{amorphous}=\frac{1}{1-X_{c}}\times (\frac{1}{M_{n}}-\frac{1}{M_{n_{0}}})$$

where *M_n_* and *M_n_*_0_ represent the number-average molecular weight and initial number-average molecular weight and *X_c_* is the crystallinity of the specimen.

## 3. Results and Discussion

### 3.1. Apparent and Microscopic Morphology of Degraded Samples

**Figure 1:**
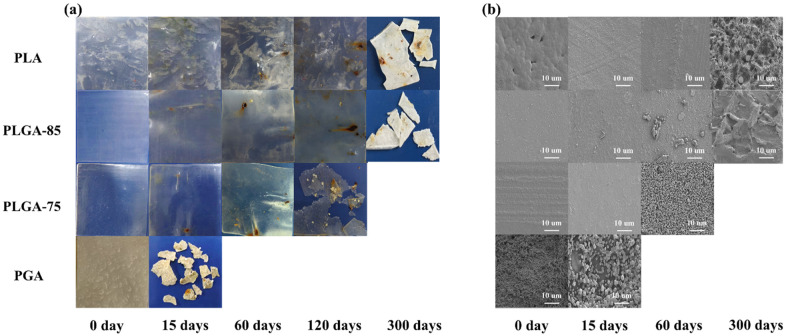
(**a**) Apparent morphology changes and (**b**) SEM images of PLA, PLGA-85, PLGA-75, and PGA before and after degradation.

The appearance changes of PLA, PLGA-85, PLGA-75, and PGA before and after degradation in seawater for several days are shown in Figure 1a. With the increase of degradation time, different samples degraded at different rates. Regarding pure PGA, its surface quickly cracked and decomposed into small fragments as the degradation proceeded. After 15 days, the PGA specimen broke completely and could not be collected, indicating it had the fastest degradation rate. Meanwhile, pure PLA underwent significant changes in the marine environment only after 120 days, turning brown with decreased transparency and toughness, eventually fracturing into small pieces. PLGA samples displayed a combination of the degradation behaviors of pure PLA and PGA. The higher the GA ratio in the blend, the faster was the degradation rate and the earlier the decomposition into small fragments. Therefore, both PLA and PGA are considered degradable in marine environment, with PGA exhibiting a more prominent degradation rate. A controllable degradation rate can be achieved by adjusting the copolymerization ratio of the two monomers.

Further studies on the microstructural evolution of PLA, PLGA-85, PLGA-75, and PGA samples before and after degradation were carried out through SEM. As shown in Figure 1b, the surface of the PGA was rougher than that of the pure PLA, PLGA-85, and PLGA-75 samples. As the degradation time increased, various degrees of corrosive cavities were observed on the sample surface, which were attributed to the water penetration and polymer chain breakage. Considering that the degradation of polyester materials usually occurred first in the amorphous region, followed by seawater gradually penetrating into the interior of the crystal, cracks and dissolution cavities appeared on the surface of the sample in the later stage of degradation. As the degradation progressed, the amorphous region continuously decreased, leaving irregular protrusions and deepening grooves. Large particles fragmented into smaller ones, accompanied by changes in crystallinity and molecular weight, as discussed in the following sections.

### 3.2. Changes in Thermal Properties and Aggregation Structure

**Figure 2:**
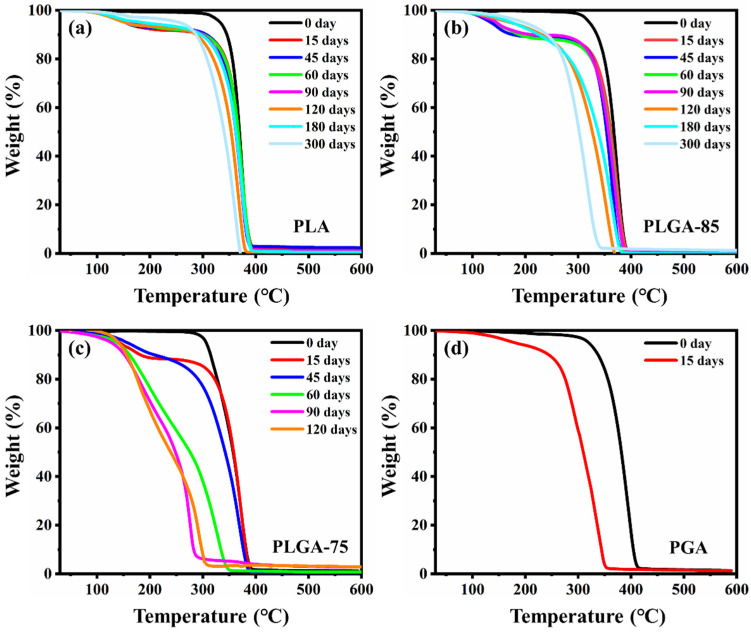
TG curves of (**a**) PLA, (**b**) PLGA-85, (**c**) PLGA-75, and (**d**) PGA before and after degradation.

Evaluation of the thermal stability of the sample was performed using thermogravimetric analysis, via measuring the initial decomposition temperature at which a 5% mass loss was observed. The weight loss curves of PLA, PLGA-85, PLGA-75, and PGA films with different degradation times are shown in Figure 2a–d. For all samples, the onset decomposition temperature (T_d_) at 5% mass loss shifted to lower temperatures after degradation, which can be attributed to the decrease in molecular weight caused by chain scission. The downward shift in the thermal decomposition temperature of PLA after degradation was less significant compared with PGA, indicating the faster chain scission of PGA. For PLGA-85 and PLGA-75, only one broad step was observed in the TG curves before degradation, while there were two distinct steps after degradation. It is speculated that this was due to the different thermal stability of different molecular chain segments in the copolymer. In the later stage of degradation, as the GA units preferentially degraded, the molecular chain segments tended to be homogeneous and the thermal stability became a broad step again. Compared with PLGA-85, the higher GA content in PLGA-75 led to a faster degradation rate and a more significant reduction in thermal stability.

**Figure 3:**
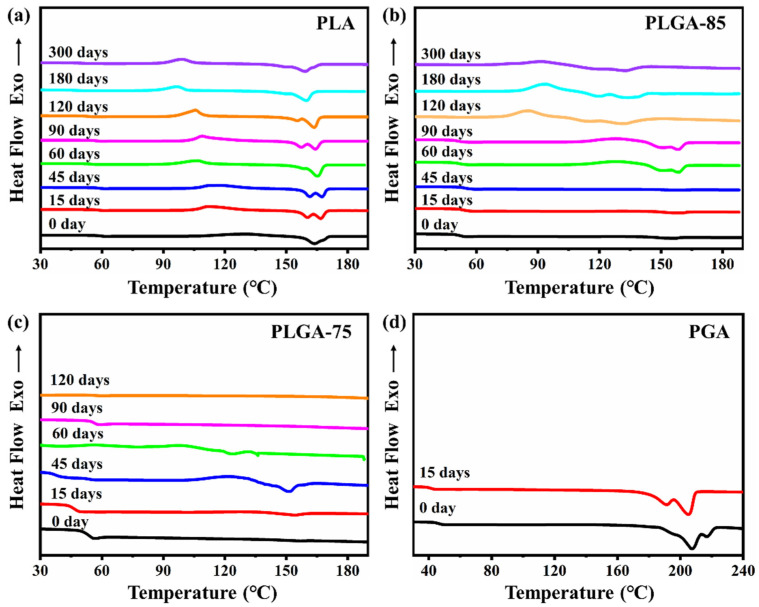
DSC second heating curves of (**a**) PLA, (**b**) PLGA-85, (**c**) PLGA-75, (**d**) PGA.

The aggregated structures of PLA, PLGA-85, PLGA-75, and PGA before and after degradation were further studied through DSC analysis. Figure 3a–d shows the DSC second-heating curves of the film samples before and after degradation. For pure PLA, the melting temperature (T_m_), cold crystallization temperature (T_cc_), and T_g_ shifted towards lower temperatures, this can also be observed from Table S1, which is believed to have been due to the enhanced mobility resulting from the breakage of molecular chains during degradation. For PGA, T_m_ and T_g_ also decreased after degradation; however, no cold crystallization was observed. Due to the different crystal structures of PLA and PGA, there were significant differences in their melting and crystallization behaviors. As for PLGA-85 and PLGA-75, no obvious crystalline peaks and melting peaks were observed in the DSC curves before degradation, indicating that neither of them were able to crystallize during the DSC experiment. As degradation progressed, the breakage of molecular chains led to cleavage-induced crystallization [37], resulting in the appearance of melting and crystallization peaks. The observed melting and crystallization peaks were close to those of PLA, indicating that the crystalline structure of the PLGA was closer to PLA when the LA content was high.

**Figure 4:**
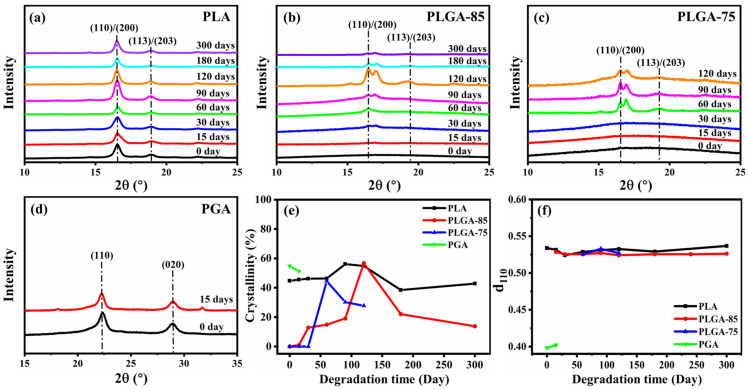
XRD curves of (**a**) PLA, (**b**) PLGA-85, (**c**) PLGA-75, (**d**) PGA, and changes in (**e**) crystallinity and (**f**) d_110_ before and after degradation.

To further investigate the evolution of crystal structure during the degradation process, XRD results are displayed in Figure 4a–f. For PLA, the diffraction peak at 2θ = 16.7° corresponded to the (110)/(200) crystal plane, while the peak at 2θ = 19.1° corresponded to the (113)/(203) crystal plane. For PGA, the diffraction peaks at 2θ = 22.4° and 2θ = 29.1° corresponded to the (110) and (020) crystal planes, respectively [38]. The diffraction peaks of PLGA-85 and PLGA-75 were similar to those of PLA, and the presence of bimodal diffraction peaks can be attributed to the coexistence of less ordered α′ and ordered α crystal forms [39]. As shown in Figure 4e, the crystallinity of PLA, PLGA-85, and PLGA-75 gradually increased in the early stage of degradation, and then continuously decreased, while the crystallinity of PGA continued to decrease with the extension of degradation time. It can be considered that PGA has a linear structure and simple repeating unit, so it directly undergoes the hydrolysis of the ester bond in seawater. In addition, due to the high GA content, it has higher hydrophilicity, making it more susceptible to erosion by water molecules, ultimately leading to faster decomposition of PGA molecular chains to reduce crystallinity. As for PLA, PLGA-85, and PLGA-75, the amorphous region degraded first in the initial stage of degradation. During this process, the fractured molecular chains reorganized to form crystalline structures, commonly referred to as cleavage-induced crystallization and typically resulting in the formation of smaller-sized defective crystalline lamellae [12,40,41]. When the degradation of the amorphous region was complete, the crystalline region began to degrade, leading to a decrease in crystallinity. In addition, there was no significant change in the interplanar distance d_110_ before and after degradation, indicating that degradation had little effect on the interlayer spacing of crystals.

### 3.3. Changes in Chemical Structure of Degraded Specimens

**Figure 5:**
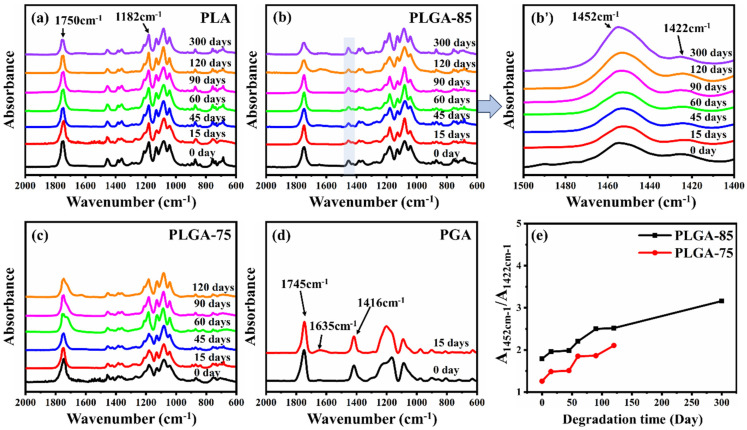
FTIR spectra of (**a**) PLA, (**b**) PLGA−85 ((**b’**) is the enlarged spectra of PLGA−85), (**c**) PLGA−75, (**d**) PGA before and after degradation, and (**e**) absorbance intensity ratio of characteristic peaks at 1452 cm^−1^ and 1422 cm^−1^ as a function of degradation time.

Chemical structural changes caused by the degradation of PLA, PLGA-85, PLGA-75, and PGA were analyzed through FTIR spectroscopy. As shown in Figure 5a–d, for pure PGA, the peak at 1745 cm^−1^ and the shoulder peak at 1635 cm^−1^ were attributed to low molecular weight esters and their free carbonyl groups, which gradually weakened and broadened during the degradation process. The peak at 1416 cm^−1^ can be attributed to the bending vibration of saturated C-H absorption in CH_2_; the peak intensity gradually decreased after degradation. The peaks at 1296 cm^−1^ are attributed to the C-O bonds of aliphatic groups, which gradually weakened during degradation and combined with C-O stretching vibration to form broad peaks. This was a result of ester bond breakage and hydrolysis forming hydroxyl radicals [42]. For PLA, the intensity of absorbance at both 1750 cm^−1^ and 1182 cm^−1^ corresponding to strong C=O stretching and C-O stretching vibration from the ester group decreased as degradation time increased. The decreased intensity reflected that the degradation of PLA occurred at the ester group in the long molecular chains [43]. For PLGA-85 and PLGA-75, the peaks at 1452 cm^−1^ and 1422 cm^−1^ represent the bending vibrations of CH_3_ and CH_2_ absorbed from LA units and GA units, respectively. To measure the relative content changes of LA and GA units during the degradation process, the absorbance intensity ratios of characteristic peaks at 1452 cm^−1^ and 1422 cm^−1^ are compared in Figure 5e. It can be seen that with the increase of degradation time, the absorbance intensity ratios of both PLGA-85 and PLGA-75 showed an increasing trend, indicating that the degradation occurred preferentially in the GA units; similar results have also been reported in other studies [44].

**Figure 6:**
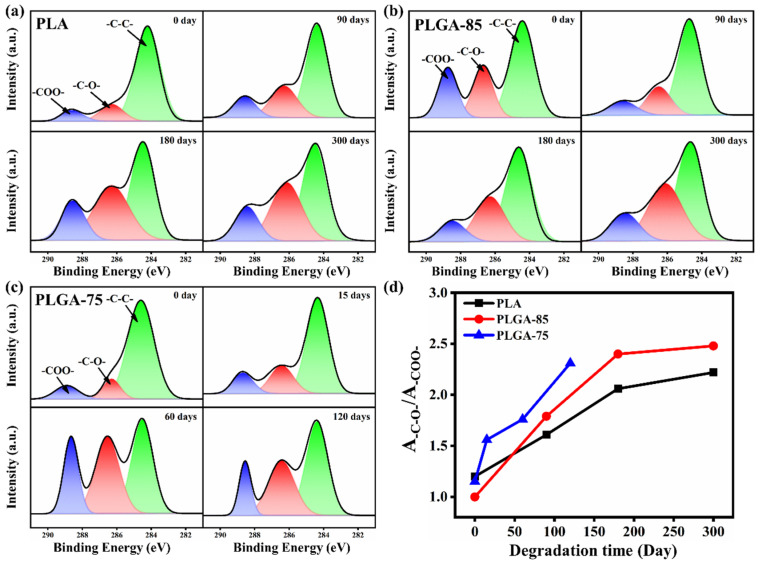
XPS C1s core-level spectra of (**a**) PLA, (**b**) PLGA-85, (**c**) PLGA-75, and (**d**) the corresponding peak area ratios of -C-O- and -COO- bonds before and after degradation.

Figure 6a–c shows the C1s core level spectra of PLA, PLGA-85, and PLGA-75 before and after degradation. The C1s core level spectra can be fitted into three peaks, with the peak values at 284.6, 286.3, and 288.6 eV representing the C-C, C-O, and -COO- groups, respectively. The peak of -COO- represents the ester bonds and carboxyl groups in the hydrolysis products, while the peak of C-O is contributed by the ester bonds, hydrolysis products, and terminal hydroxyl groups. To evaluate the degradation rates, the relative peak area ratios of C-O to -COO- are compared in Figure 6d. The corresponding peak area ratios of PLA and PLGA-85 increased from 1.3 to 2.22 and 1.0 to 2.48 after 300 days, while the value of PLGA-75 increased from 1.15 to 2.31 after 120 days. Since a larger relative peak area ratio indicates a higher degradation rate in the film samples, it can be concluded that the degradation rate of PLGA-75 was the highest, whereas the degradation rate of PLA was the lowest.

### 3.4. Changes in Molecular Weight and Polydispersity Index of Degraded Specimens

**Figure 7:**
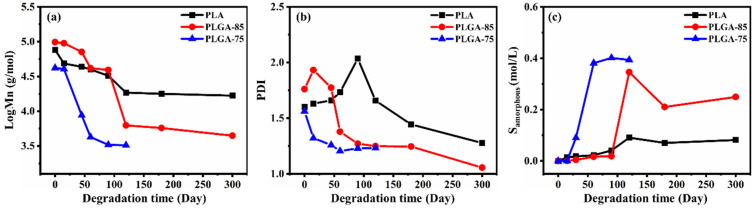
Changes in the (**a**) number-average molecular weight (*M_n_*), (**b**) polydispersity index (PDI), and (**c**) chain scission concentration in the amorphous phase of PLA, PLGA-85, and PLGA-75 as a function of degradation time.

Since PGA is insoluble in conventional organic solvents, only the number-average molecular weight (*M_n_*) and polydispersity index (PDI) of PLA, PLGA-85, and PLGA-75 during degradation were measured via GPC. As shown in Figure 7a, the *M_n_* values of these three samples all decreased after degradation, and PLGA-75 decreased the fastest, followed by PLGA-85, and then PLA. In a marine environment, the LA/GA ratio determines the extent of *M_n_* changes. A higher proportion of LA units in the polymer main chain slows down hydrolysis, while GA units can promote hydration and the diffusion of water, thereby accelerating the hydrolysis process [45,46,47]. Regarding the PDI values shown in Figure 7b, those of PLA and PLGA-85 increased first and then decreased during degradation, while the values of PLGA-75 continuously decreased, the molecular weight distribution curve of Figure S1 also demonstrates this point. These results indicate that the higher GA content of PLGA-75 can cause a more uniform breakage of molecular chains. In addition, the concentration of chain scission in the amorphous phase (S_amorphous_) is further compared in Figure 7c. When hydrolysis occurred, chain breakage first took place in the amorphous phase due to the looser molecular chains facilitating the entry of water. For all these three samples, the values of S_amorphous_ during degradation all showed a trend of initially increasing and then maintaining a constant. Among them, at the same degradation time, PLGA-75 possessed the largest value, followed by PLGA-85, and PLA displayed the smallest value, which further confirmed that higher GA content led to a faster degradation rate.

### 3.5. Structural Evolution Discussion

**Figure 8:**
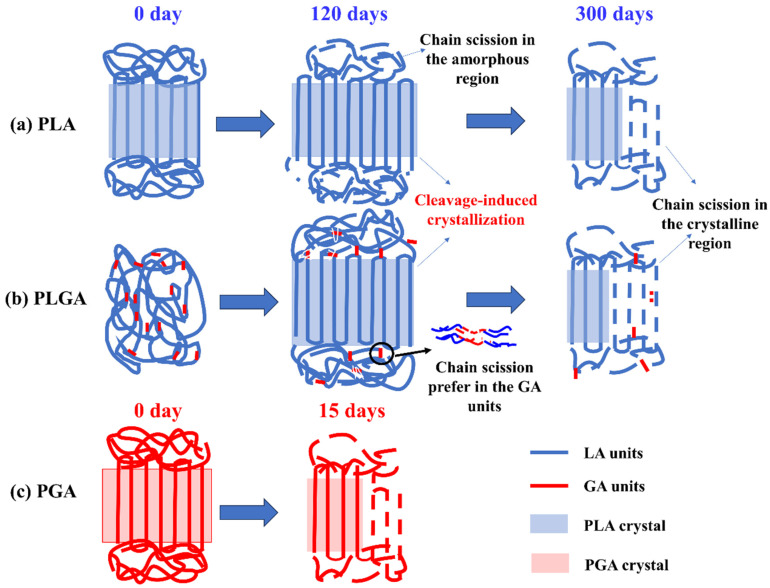
Schematic models of microstructure evolution during degradation in a marine environment: (**a**) PLA, (**b**) PLGA, (**c**) PGA.

Summarizing the above TG, DSC, XRD, FTIR, XPS, and GPC results, the structural evolution of PLA, PLGA, and PGA films during hydrolysis were analyzed. The schematic diagram shown in Figure 8 describes the structural changes during degradation. The degradation of PLA, PGA, and PLGA in the simulated marine environments mainly involved the hydrolysis of ester bonds. For PLA, the chain scission caused by hydrolysis first occurred in the amorphous region, and due to the enhanced mobility of fractured molecular chains, cleavage-induced crystallization was generated. As the degradation time extended, the crystalline region gradually underwent degradation, generating fragments with low molecular weight. Therefore, during the degradation process, the molecular weight of the PLA gradually decreased, while the crystallinity first increased and then decreased. The PGA, due to its linear structure and simple repeating units, directly underwent hydrolysis of the ester bond in seawater. In addition, due to its higher hydrophilicity than PLA, the PGA molecular chains decomposed faster, thereby reducing the crystallinity. As for the copolymers, due to the superior hydrophilicity of GA units compared with LA units, the hydrolysis of GA units took place preferentially, and cleavage-induced crystallization also appeared, making the crystallinity first increase and then decrease. Regarding the PLGA-85 and PLGA-75 with high LA content, their crystal structures were similar to PLA and contained less ordered α′ and ordered α crystal forms. In addition, the higher the GA content, the faster was the degradation rate of PLGA.

## 4. Conclusions

In this work, the degradation behaviors of PLA, PGA, and their copolymers PLGA-85 and PLGA-75 in simulated marine environments were investigated. Polyester materials underwent degradation in marine environments primarily through the hydrolysis of ester bonds, with PGA degrading faster than PLA. During the degradation process, the molecular chains broke, leading to a decrease in molecular weight and a reduction in the thermal stability of the materials. The crystal structures of PLGA-85 and PLGA-75 were similar to that of PLA, and the higher the GA unit content, the faster was the degradation rate, which could be attributed to the superior hydrophilicity of GA units compared with LA units. Meanwhile, PGA, due to its linear structure and simple repeating units, directly underwent hydrolysis of the ester bond in seawater and the molecular chains broke down to reduce the crystallinity. This study provides a deeper understanding of the seawater degradation behaviors of PLA, PGA, and their copolymers, and provides guidance for the preparation of materials with controllable degradation performance.

## Data Availability

Data are contained within the article and Supplementary Materials.

## Supplementary Materials

The following supporting information can be downloaded at: https://www.mdpi.com/article/10.3390/polym16131765/s1, Figure S1: Signal strength versus retention time change curves (a) PLA, (b) PLGA-85, (c) PLGA-75; Table S1: Changes of Tg, Tcc, and Tm during different degradation times.
