# Supplementary material for: Degradation Behaviors of Polylactic Acid, Polyglycolic Acid, and Their Copolymer Films in Simulated Marine Environments
Source: Polymers (Basel). 2024 Jun 21;16(13):1765. doi: 10.3390/polym16131765 (PMC11244091; doi:10.3390/polym16131765)
Supplement: Supplementary file 1 [file polymers-16-01765-s001.zip › polymers-3030047-supplementary.pdf]

Supporting Information

Degradation Behaviors of Polylactic Acid, Polyglycolic Acid, and Their Copolymer Films in Simulated Marine Environments

Zeyu Chen [1], Xi Zhang [1,*], Ye Fu [1], Yujuan Jin [1], Yunxuan Weng [1,*], Xinchao Bian [2] and Xuesi Chen [2]

[1] College of Light Industry Science and Engineering, Beijing Technology and Business University, Beijing 100048, China; c1554192404@163.com (Z.C.); fuye@btbu.edu.cn (Y.F.); jinyujuan@th.btbu.edu.cn (Y.J.)
[2] Changchun Institute of Applied Chemistry, Chinese Academy of Sciences, Changchun 130022, China; xcbian@ciac.ac.cn (X.B.); xschen@ciac.ac.cn (X.C.)
* Correspondence: zhangxi2021@btbu.edu.cn (X.Z.); wyxuan@th.btbu.edu.cn (Y.W.)

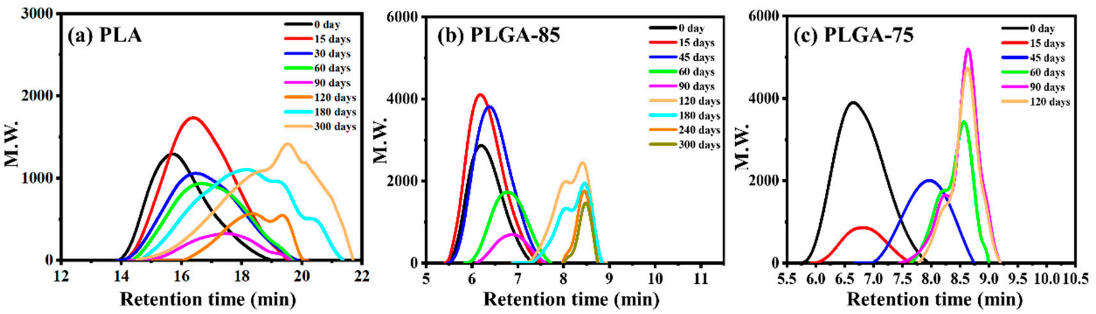

Figure S1. Signal strength versus retention time change curves (a) PLA, (b) PLGA-85, (c) PLGA-75.

Table S1. Changes of T_g, T_{cc}, and T_m during different degradation times.

				T_g (°C)				
Degradation time (Day)	0	15	45	60	90	120	180	300
PLA	59.08	58.75	57.59	56.39	55.63	54.24	52.88	51.12
PLGA-85	55.26	54.10	53.11	51.95	50.79	49.59	48.63	47.64
PLGA-75	52.21	45.74	40.27	38.91	37.16	36.96		
PGA	40.14	36.79						
				T_{cc} (°C)				
Degradation time (Day)	0	15	45	60	90	120	180	300
PLA	131.09	112.2	112.74	107.04	109.03	105.63	96.55	98.47
PLGA-85	—	—	—	128.81	129.38	84.81	93.20	91.84
PLGA-75	—	—	122.06	101.34	97.40	89.37		
PGA	—	—						
				T_m[a] (°C)				
Degradation time (Day)	0	15	45	60	90	120	180	300
PLA	163.78	159.89	161.28	158.92	157.17	155.61	151.10	150.51
PLGA-85	—	155.58	152.26	150.70	150.30	131.73	120.39	119.79
PLGA-75	—	154.35	151.60	122.66	118.08	112.88		
PGA	207.33	191.11						

a. T_m values correspond to the peak temperature of the low-melting peak.
